# Pharmacologically Active Phytomolecules Isolated from Traditional Antidiabetic Plants and Their Therapeutic Role for the Management of Diabetes Mellitus

**DOI:** 10.3390/molecules27134278

**Published:** 2022-07-03

**Authors:** Prawej Ansari, Samia Akther, J. M. A. Hannan, Veronique Seidel, Nusrat Jahan Nujat, Yasser H. A. Abdel-Wahab

**Affiliations:** 1 Department of Pharmacy, Independent University, Dhaka 1229, Bangladesh; samiaakther147@gmail.com (S.A.); jmahannan@iub.edu.bd (J.M.A.H.); njnujat@gmail.com (N.J.N.); 2 School of Biomedical Sciences, Ulster University, Coleraine BT52 1SA, UK; y.abdel-wahab@ulster.ac.uk; 3 Natural Products Research Laboratory, Strathclyde Institute of Pharmacy and Biomedical Sciences, University of Strathclyde, Glasgow G4 0RE, UK; veronique.seidel@strath.ac.uk

**Keywords:** medicinal plants, traditional medicine, phytoconstituents, diabetes, pharmacology

## Abstract

Diabetes mellitus is a chronic complication that affects people of all ages. The increased prevalence of diabetes worldwide has led to the development of several synthetic drugs to tackle this health problem. Such drugs, although effective as antihyperglycemic agents, are accompanied by various side effects, costly, and inaccessible to the majority of people living in underdeveloped countries. Medicinal plants have been used traditionally throughout the ages to treat various ailments due to their availability and safe nature. Medicinal plants are a rich source of phytochemicals that possess several health benefits. As diabetes continues to become prevalent, health care practitioners are considering plant-based medicines as a potential source of antidiabetic drugs due to their high potency and fewer side effects. To better understand the mechanism of action of medicinal plants, their active phytoconstituents are being isolated and investigated thoroughly. In this review article, we have focused on pharmacologically active phytomolecules isolated from medicinal plants presenting antidiabetic activity and the role they play in the treatment and management of diabetes. These natural compounds may represent as good candidates for a novel therapeutic approach and/or effective and alternative therapies for diabetes.

## 1. Introduction 

Diabetes mellitus is one of the most common endocrine metabolic disorders characterized by chronic hyperglycemia caused by varying degrees of insulin resistance, deficiency in insulin secretion, or both [1]. Nearly 10.5% of the worldwide population is affected by diabetes, with its prevalence increasing at an alarming rate. According to data collected from the International Diabetes Federation (IDF), about 783.2 million people are estimated to be diagnosed with diabetes by 2045 [2]. Diabetes mellitus can be classified into two major categories: Type 1 and Type 2 diabetes, where Type 2 diabetes accounts for about 90% of all cases. Type 1 diabetes, previously known as insulin-dependent diabetes, is an autoimmune disorder that occurs due to the destruction of the pancreatic beta cells leading to significantly reduced secretion of insulin [3]. It is a non-hereditary genetic condition that mainly affects the juvenile under thirty years of age. Type 2 diabetes, also known as non-insulin-dependent diabetes, is the most common form of diabetes, with its prevalence rapidly rising worldwide [4]. It is a hereditary condition caused as a result of insulin resistance, insufficient insulin secretion, or a combination of both, largely affecting an older population than Type 1 diabetes [5]. Both forms of diabetes alter carbohydrate, protein, and fat metabolism. The effect of insulin resistance leads to high blood sugar levels by hindering the uptake and efficient use of glucose by most cells of the body [6]. The progression of the disease is accompanied by tissue or vascular damage resulting in severe complications, including retinopathy, diabetic neuropathy, nephropathy, cardiovascular, pulmonary, cerebral, and peripheral vascular diseases, ulcers, and thyroid gland disorders, leading to serious morbidity and mortality [1,7,8,9]. Available therapies currently in use for the treatment and management of diabetes include insulin and several oral hypoglycemic agents such as metformin, sulfonylureas, α-glucosidase inhibitors, meglitinide analogues, thiazolidinediones, DPP-IV inhibitors, SGLT-2 inhibitors, and GLP-1 mimetics. However, these drugs, intended to boost insulin sensitivity and increase insulin secretion together with the reduction in circulatory plasma glucose levels by increasing glucose excretion or uptake in adipose tissue, are usually associated with many side effects. These include, among others, weight gain, hypoglycemia, gastrointestinal tract disturbances, liver injury, renal failure, hypersensitivity reactions, flatulence, diarrhea, and abdominal bloating [1,10,11]. In addition, these drugs have been known to have other major disadvantages, including drug resistance, and there is also a lack of therapies to prevent the long-term complications of the disease. 

The complications associated with insulin and oral antidiabetic agents, together with limited drug tolerability, adverse effects, and cost, have accelerated the search for alternative medicines with better efficacy, potency, and fewer side effects [12]. Interestingly, there has been an increase in popularity surrounding drug discovery research into natural antidiabetic agents, especially those derived from medicinal plants, which could enhance β-cell function and treat diabetes-associated complications with fewer adverse side effects [13]. 

Herbal medicines contain a diversity of phytochemicals and have been traditionally used for treating a wide variety of diseases. They are considered to be naturally safe and efficacious with fewer side effects [12]. The control and management of diabetes using herbal drugs have proven to be more advantageous over synthetic medicines due to their accessibility, reduced cost, lesser complications, and lower side effects. Herbal medicines act via different mechanisms aiming at reducing insulin resistance, increasing insulin secretion, protecting pancreatic beta cells, and thereby lowering circulating blood glucose levels [14]. 

Throughout the years, thousands of plant species have been used for their medicinal uses as integrative medicines for various diseases, of which more than 800 plants have been reported to exhibit antidiabetic effects [15]. Such plants have been examined for their use in the treatment of the different types of diabetes and could be potential sources for new natural antidiabetic drug discovery research [16]. A number of medicinal plants used traditionally for their antidiabetic activity are currently under investigation to be formulated commercially as modern drugs. This is particularly the case in developing countries where the cost of allopathic medicine is high, and the traditional use of plants to treat diabetes is common practice [15]. Traditional natural medicines are extensively prescribed in Asian countries (e.g., China, India, Bangladesh, Pakistan, Sri Lanka, Thailand, Nepal, Bhutan, Japan, and others) [17]. Among the medicinal plants possessing hypoglycemic effects, the most common ones used as remedies for diabetes include *Acacia arabica*, *Aegle marmelos*, *Allium cepa*, *Allium sativum*, *Aloe vera*, *Annona squamosa*, *Azadirachta indica*, *Berberis vulgaris*, *Camellia sinensis*, *Capsicum frutescens*, *Cassia alata*, *Cinnamomum zeylanicum*, *Eucalyptus globulus*, *Eugenia jambolana*, *Helicteres isora*, *Momordica charantia*, *Panax ginseng*, *Punica granatum*, *Swertia chirayita*, *Trigonella foenum-graecum*, and others [15,16,18,19]. The antidiabetic activity of these plants is thought to be mediated via various mechanisms, including the stimulation of insulin secretion from pancreatic β-cells, increasing insulin binding to receptors, reduction in insulin resistance, and improving glucose tolerance. Other modes of action include enhancing glucose metabolism, improving β-cell mass and function, and increasing plasma insulin, thus decreasing circulating blood glucose levels [20,21,22,23]. In addition to being used to treat diabetes, these plants have also been traditionally employed to treat other conditions such as ulcers, wounds, inflammation, infections, diarrhea, dysentery, malaria, rheumatism, hypertension, obesity, pneumonia, and kidney diseases [12,19,24,25,26]. The main objective of this review is to explore the traditional plant-based therapies and/or their phytoconstituents available for the treatment of diabetes. These could provide the basis for the discovery of new antidiabetic drugs with fewer side effects and stronger efficacy than currently available medicines.

## 2. Methods

A literature search was carried out via Google Scholar, ScienceDirect, Scopus, and PubMed databases to accumulate data for this review article using the keywords “Diabetes mellitus,” “Medicinal Plants,” “Traditional medicine,” “Antidiabetic phytochemicals,” and “Plant-based antidiabetic therapy.” The data search was not restricted to a specific time period; however, around 98% of the gathered data were published between 2000 and 2022, and only 2% were published before 2000. Our data collection began in early January until late May 2022. More than 700 papers were found relevant to our study, and after performing a primary screening, around 400 papers were selected to be critically examined. An overview of the key findings has been presented in this current review.

## 3. Ethnomedicines and Their Scope in the Modern World

Ethnomedicine is a traditional health care practice followed by indigenous people concerned with human health. It is the origin of all other traditional medical systems, including Ayurveda, Siddha, Unani, Nature Cure, as well as modern medicine [27]. Knowledge of plants presenting therapeutic properties has been passed on by experimenting through trials and errors from one generation to the next for more than hundreds of years. Ethnomedicines are highly prevalent in the rural and native communities of several developing countries [28]. According to information collected from the World Health Organization, about 80% of the global population relies upon traditional remedies [29]. Medicinal plants have always been recognized as a major source of raw materials for both conventional and traditional medicines [30]. In India, the poor and rural residents are dependent upon natural herbal remedies since they are easily obtainable to them. Indeed, plant-based medicines are the sole source of medical management for people living in remote areas. In countries such as Russia, Africa, and a few European countries, ethnomedicines are being studied by various botanists, anthropologists, folklorists, and medical scientists [27]. The inability for people to access adequate healthcare, alongside financial restrictions, has resulted in the under-provision of modern health care for a majority of the people in underdeveloped countries. [31]. Numerous folk remedies are recorded as being effective in treating various diseases (such as digestive tract disorders, skin diseases, renal and liver diseases, malaria, ulcers, heart diseases, pneumonia, diabetes, and many others), and thus, even developed countries have also considered utilizing these medicines [32]. 

## 4. Plant-Based Medicine versus Synthetic Medicine

Many drugs that are currently available have been derived directly or indirectly from natural sources such as medicinal plants and animals [33,34]. Plant-derived natural products have played and continue to play a prominent role in drug discovery and development programs. The increase in the number of herbal drug manufacturing companies, linked to the current increase in interest and demand for herbal medicines, can be largely expanded because of the toxicity and numerous adverse effects of allopathic medicines [35]. The convenience of accessibility, availability, inexpensiveness, and relatively low risks of side effects, have caused plant-based medicines to be an important alternative source of existing therapies, especially in rural and/or developing regions [33]. Plant-based medicines also provide a rich source of biologically active compounds that possess pharmacological activity with minimal undesirable effects [33]. 

Over the centuries, plant-based medicines have been widely used to treat the ailments of local communities of many developing countries that have easy access to these sources. Densely populated countries, such as China and India, have especially contributed to the advancement of sophisticated traditional medical systems such as acupuncture, ayurvedic medicine, and herbal medicine [36]. Many factors should be considered when selecting the appropriate medications for the management and treatment of diabetes. This includes efficacy, adverse effects, cost, and potential to contribute to weight gain, risks associated with hypoglycemia, comorbidities, and patient compliance. Even though oral antihyperglycemic agents can lower plasma glucose levels by improving insulin secretion or reducing insulin resistance, they are associated with many other adverse effects. Metformin, the mainstay of treatment in type 2 diabetes, has a high safety profile, yet it is still associated with mild side effects such as low risks of hypoglycemia and gastrointestinal tract disturbances (nausea, diarrhea, dyspepsia). Previous studies have shown that continuous use of metformin may result in vitamin B12 and folic acid deficiency in humans [37]. DPP-IV inhibitors such as sitagliptin, saxagliptin, and linagliptin, have been found to cause headaches, nasopharyngitis, and upper respiratory tract infections [38]. The most common adverse effect of sulphonylureas such as glimepiride and gliclazide is hypoglycemia. These drugs are also associated with minor side effects such as weight gain, nausea, headaches, drowsiness, and hypersensitivity reactions. The most serious complication of insulin injections is hypoglycemia. Insulin may also cause weight gain or loss, dizziness, confusion, and sweating [38]. In contrast to synthetic drugs, plant-based medicines do not interrupt the body’s natural healing process; instead, they accelerate the recovery process by strengthening the healing process, ultimately leading to a steady recovery. Alongside their ability to help the body recover to a healthy status, herbal medicines are also known for boosting the immune system. The use of highly effective herbal medicines showing fewer side effects and a strong immune system together with a healthy lifestyle promotes better body metabolism with increased nutritional absorption from the diet [35]. Whether they have insulinotropic, insulin-mimetic, or any other antihyperglycemic effects, medicinal plants are considered safer and more effective alternatives to synthetic antidiabetic drugs [39].

## 5. Pharmacological Activity of Plant-Based Medicines

Although knowledge of many plant-based therapies has been transmitted through generations, only a few of these have started to come to the fore recently. However, there is still some uncertainty regarding their pharmacological activity as well as their acute/chronic side effects due to such medicines being broadly underreported [40]. Few plants have proven to be efficacious for which they were intended, whilst some were not strongly therapeutically effective and/or sufficient scientific data were lacking to support their expected effects [41]. The increase in the widespread use of plant-based therapies has led to an urgent need for a detailed scientific examination of the chemicals responsible for pharmacological activity. Indeed, such a study of the pharmacological properties and phytoconstituents of plant-based medicines may lead to the discovery of new pharmacological characteristics previously unknown or used in traditional medicine [42]. Herbal medicines have been suggested to exert their mechanism of action by concurrently targeting multiple physiological processes via interactions between different biochemicals and cellular proteins [43]. 

**Figure 1:**
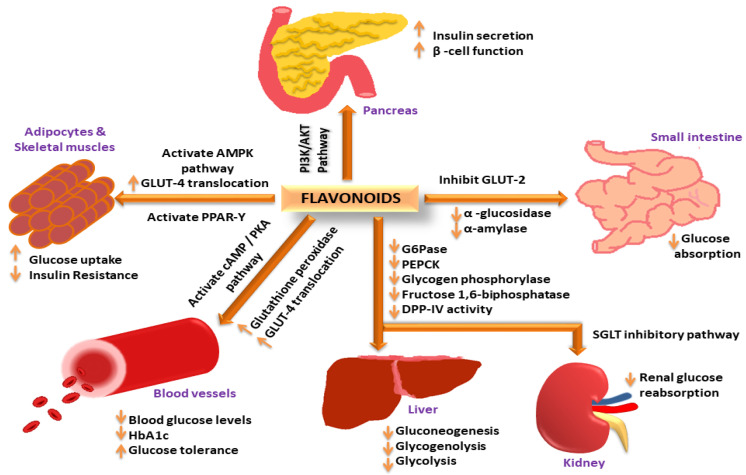
Flavonoids exerting antidiabetic activity via different mechanistic pathways: Flavonoids increase insulin secretion and improve β-cell function via the PI3K/AKT signaling pathway; increase GLUT-4 translocation through AMPK activation to increase glucose uptake in adipose tissues and skeletal muscles; activate PPAR-γ expression to decrease insulin resistance; activate cAMP/PKA pathway to reduce blood glucose levels and improve glucose tolerance; increase glutathione peroxidase activity to reduce HbA1c levels; decrease G-6-Pase, PEPCK, glycogen phosphorylase, fructose 1,6-biphosphatase and DPP-IV activity in liver to decrease gluconeogenesis, glycogenolysis, and glycoslysis; inhibit SGLT pathway in kidney to decrease renal glucose reabsorption; inhibit GLUT-2, α-amylase and α-glucosidase activity to decrease glucose absorption in the small intestine.

Herbal medications may be able to alter the biological systems from disease to a healthy state by causing the interactions between multi-component and multi-target. Because of the therapeutic properties of the phytomolecules, a lower dosage may be used, resulting in less toxicity and adverse effects. [43]. The antidiabetic activity of medicinal plants is dependent upon the phytochemicals that act through multiple pathways, such as cAMP: which stimulates insulin secretion without affecting the K_ATP_ channel [44]; PI3K: which facilitates glucose uptake by the translocation of the glucose transporter in skeletal muscles, adipose tissue, or liver [45]; AMPK: The activation of 5ʹ-adenosine monophosphate-activated protein kinase pathway improves insulin sensitivity by limiting lipolysis and lipogenesis, and AMPK also enhances glucose uptake in skeletal muscles by translocating GLUT4-containing intracellular vesicles across the plasma membrane [46,47]. For example, phlorizin obtained from the bark of apple and pear trees increases glucose excretion in urine by decreasing glucose reabsorption in the kidneys via the inhibition of SGLT and thus, lowers plasma glucose concentration [48]. Some of the phytomolecules have the potential to regenerate and protect pancreatic beta cells from destruction by reducing the glucose load [49], inhibiting α-amylase and α-glucosidase activity, inducing glucose uptake in 3T3L1 cells [50,51], inhibiting aldose reductase enzyme activity, glycogen metabolizing enzymes, exerting hepato-pancreatic protective activity, inhibiting glucose-6-phosphate and DPP-IV, reducing lactic dehydrogenase, γ-glutamyl transpeptidase, glycosylated hemoglobin levels, and inhibiting glycogenolysis and gluconeogenesis in the liver [20,52]. As an example, a summary of the different pathways involved in the antidiabetic activity of flavonoids is illustrated in Figure 1. A summary of antidiabetic medicinal plants and their pharmacological actions has been shown in Table 1.

## 6. Phytochemicals and Their Impact on Diabetes

Plants are the primary source of biologically active compounds that may ultimately lead to the discovery and development of potential new drugs [238]. Plants produce both primary and secondary metabolites. Carbohydrates, proteins, and lipids are considered primary metabolites, necessary for the growth and development of plants and involved in essential metabolic pathways, such as photosynthesis and glycolysis. Secondary metabolites are not required for the growth and development of plants; rather, they are responsible for interactions between plant species and the environment and have highly specific functions in plants [239]. 

Over 13,000 secondary metabolites have been purified and isolated from medicinal plants. These phytochemicals can be categorized into various chemical classes such as alkaloids, flavonoids, terpenoids, phenolics, tannins, saponins, xanthones, and glycosides [78]. Many of these phytochemicals are known to exhibit medicinal properties, including antidiabetic activity [78]. Several phytochemicals isolated from various plant species have been scientifically validated for their contribution to treating and managing diabetes by exerting antihyperglycemic activity and reducing the complications associated with diabetes [171]. For example, the flavonoid rutin, present in the leaves of numerous plants, including *Annona squamosa* and *Azadirachta indica* (neem), has been reported to possess many beneficial effects such as anti-inflammatory, anti-cancer, anti-allergic, antiviral, and antioxidative properties [240]. Rutin-containing plants have also been shown to protect against heart disease, hepatotoxicity, and diabetes mellitus [240]. Rutin exerts its antidiabetic effect by lowering plasma glucose, improving the function of pancreatic β-cells, and enhancing glucose tolerance [10]. Two other flavonoids found in the leaves of *Annona squamosa*, namely quercetin and isoquercetin, have also been reported to possess antihyperglycemic activity by inhibiting α-glucosidase and lowering blood glucose levels [241]. Alongside rutin and quercetin, the tetranortriterpenoid meliacinolin, isolated from the leaves of *A. indica,* has been found to inhibit α-glucosidase and α-amylase in Type 2 diabetic mice [98]. Nimbidin, extracted from neem seeds, is another phytochemical exhibiting hypoglycemic properties [98]. Quercetin, allicin, allyl-propyl disulfide, cysteine sulfoxide, and S-allyl cysteine sulfoxide from *Allium sativum* (garlic) have been reported to stimulate insulin secretion from pancreatic β-cells, increase insulin sensitivity to target cells, and prevent insulin activation triggered by the liver [71]. Alliin, from garlic, has been reported to mimic the function of glibenclamide and insulin [71]. Epigallocatechin-3-gallate, epigallocatechin, epicatechin-3-gallate, and epicatechin present in *Camellia sinensis* (tea) leaves can also lower plasma glucose levels by improving β-cell function, increasing insulin secretion, and enhancing glucose metabolism [117]. These phytomolecules may exert their antidiabetic activity in multiple manners, most commonly by being insulinotropic, insulin-mimetic, and by improving β-cell function, increasing insulin sensitivity, improving glucose tolerance and metabolism, as well as inhibiting various enzyme activities. A summary of antidiabetic medicinal plants and their phytochemicals with potential antidiabetic effects is provided in Table 2. The chemical structures of the antidiabetic phytoconstituents of medicinal plants are given in Table 3.

## 7. Plant-Based Drug Formulations Available on the Market and Their Role in Diabetes

For the past few decades, there has been an increasingly growing trend in many European countries to develop and sell plant-based medicines [370]. The latter are known as herbal formulations or phytomedicines. These preparations have been standardized and confirmed for their safety profile and effectiveness in the treatment of various diseases. Similar to any other allopathic medicine, herbal formulations can also be prepared as diverse formulations such as tablets, capsules, elixirs, suspensions, solutions, emulsions, and powders [371]. Phytomedicines can either be single herb- or polyherbal formulations [35]. Several phytomedicines have been marketed worldwide for the control and management of diabetes. These include Antibetic, Diabetics, Diabetica, Diabet, Diasol, Diabecon, Diasulin, Dia-Care, Diabecure, Diabeta, Diabeta Plus, Dianex, Diashis, GlucoCare, GlycoNase, Glyoherb, Karmin Plus, SugarMax, and Sugar Loss [35,372]. These products comprise a combination of individual constituents from several antidiabetic plants. Many of these preparations are sold with directions about diet, rest, and physical activities to enhance their effectiveness [35,372]. 

## 8. The Future of Plant-Based Antidiabetic Medicines

Nearly 75% of the globally used herbal medicines have been developed based on traditional medicine practitioners [24]. Medicinal plants will continue to be used for their natural safety and potency in many remedies, as well as cosmetics, perfumes, and in the food and beverages industry [373]. Biologically active components derived from traditional medicinal plants have yielded several clinically used drugs and still play a key role in the discovery of new medicines. Thus, it is reasonable to assume that plants used in folk medicine can be used as a potential source for the discovery of new drugs to treat diabetes. The most frequently recommended synthetic drug, metformin, has blood glucose-lowering properties in Type 2 diabetes and the search for many such drugs persists [370]. Moreover, any plant-derived antidiabetic drug with a novel mode of action compared to existing antidiabetic agents has a high potential to be used in clinics [374]. Although the use of plant-based medicines is widespread in developing countries, recently, developed countries have also shown interest in using herbal drugs and therapies. With the rise in the incidence of diabetes mellitus, the demand for plant-based antidiabetic medicines is increasing worldwide. It is expected that countries such as China, India, and Japan, which have an abundance of medicinal plant species and are the greatest exporters of medicinal plants worldwide, will be the most sought [375]. More studies are required regarding the pharmacokinetics/pharmacodynamics of different phytoconstituents in laboratory animals and in clinical use to establish the benefits and mode(s) of action of these compounds in the treatment and management of diabetes. Extensive investigations into the pharmacology, toxicology, metabolism, and tissue distribution of medicinal plants and their phytomolecules are necessary for the development of new potent antidiabetic drugs [376].

## 9. Conclusions

Diabetes mellitus has risen as a major public health crisis, particularly in underdeveloped countries. Thus, recent research efforts have been centered on the discovery of new natural sources of antidiabetic therapies for the treatment and management of diabetes. As traditional medicinal plants with antidiabetic activity may be considered potential candidates for diabetes management in the long run, they are being extensively researched for novel targets, mechanisms of action, and routes of administration. Plant-based antidiabetic medicines are inexpensive, readily available, and hold low risks of side effects. This makes them promising new antidiabetic agents. With the progression of medicinal plant-based research, scientists and physicians have started to develop newer classes of antidiabetic drugs based on the pharmacology of the phytochemicals isolated from these plants. However, more studies are required for in-depth investigation of these newly discovered antidiabetic drugs at the molecular, therapeutic, and physiological levels in order to control and manage diabetes mellitus worldwide.

## Figures and Tables

**Table 1:** Traditional uses and pharmacological effects of antidiabetic medicinal plants.

Medicinal Plants	Parts	Traditional Uses	Pharmacological Effects	References
*Abrus precatorius*	Leaves, seeds	Diabetes, wounds, fever, cough, cold, tetanus	Improves β-cell function, inhibits α-amylase and α-glucosidase activity	[53,54]
2. *Acacia arabica*	Bark, roots	Diabetes, astringent, diarrhea, parasitic worms, diuretic, liver tonic	Lowers blood glucose levels, increases insulin secretion, improves glucose uptake and glucose tolerance	[24,55]
3. *Acacia catechu*	Bark	Diabetes, asthma, bronchitis, diarrhea, obesity, dysentery, skin diseases	Lowers blood glucose levels, increases insulin secretion	[56,57,58]
4. *Aegle marmelos*	Leaves	Diabetes, dysentery, inflammation, ulcer, diarrhea, asthma	Lowers blood glucose levels, increases insulin secretion, glucose uptake and metabolism, inhibits aldose reductase and DPP-IV enzyme activity	[56,59,60]
5. *Aframomum melegueta*	Fruit, leaves	Diabetes, cough, diarrhea, stomach ache, leprosy, hypertension, measles	Lowers plasma glucose levels, inhibits α-amylase and α-glucosidase activity	[61,62]
6. *Ageratum conyzoides*	Leaves	Diabetes, fever, rheumatism, cardiovascular diseases, malaria, wounds, spasms	Lowers blood glucose levels, improves β-cell function, increases insulin secretion	[63,64]
7. *Albizia lebbeck*	Bark, pods	Diabetes, asthma, diarrhea, infections, dysentery, inflammation	Lowers blood glucose levels, increases insulin secretion, enhances glucose uptake	[56,65,66]
8. *Albizia adianthifolia*	Bark, leaves	Diabetes, eye problems, hemorrhoids, skin diseases, wounds, malaria diarrhea, indigestion	Lowers blood glucose levels, improves glucose tolerance	[16,67]
9. *Allium cepa*	Bulb	Diabetes, bronchitis, hypertension, skin infections, swelling, lower cholesterol level	Increases insulin secretion and insulin sensitivity, improves glucose uptake	[68,69]
10. *Allium sativum*	Bulb	Diabetes, fever, hypertension, rheumatism, dysentery, bronchitis, intestinal worms	Increases insulin secretion and insulin sensitivity to cells	[70,71]
11. *Aloe vera*	Leaves	Diabetes, constipation, infections, ulcer, dysentery, piles, rheumatoid arthritis	Lowers blood glucose levels, increases insulin secretion, reduces insulin resistance, improves glucose tolerance	[72,73]
12. *Anacradium occidentale*	Leaves, stem bark	Diabetes, fever, hypertension, rheumatism, toothache, piles, dysentery	Lowers blood glucose levels, reduces oxidative stress, decreases total cholesterol and triglyceride levels	[74,75,76]
13. *Anemarrhena asphodeloides*	Rhizome	Diabetes, fever, cough, inflammation, infections, night sweats, dementia	Lowers blood glucose levels, increases insulin sensitivity, improves glucose uptake	[77,78]
14. *Annona salzmannii*	Leaves, bark	Diabetes, inflammation, tumors	Lowers blood glucose levels, improves β-cell function, increases insulin secretion	[79,80]
15. *Annona squamosa*	Leaves	Diabetes, wounds, inflammation, hypertension, malaria, insect bites	Lowers blood glucose levels, increases insulin secretion, improves glucose tolerance and β-cell function	[10,81]
16. *Anogeissus latifolia*	Bark	Diabetes, diarrhea, hemorrhoids, dysentery, snake bites, stomach disorders, skin diseases, leprosy	Decreases blood glucose levels, improves β-cell function, increases insulin secretion, inhibits DPP-IV enzyme activity	[56,82,83]
17. *Arachis hypogaea*	Seeds	Diabetes, inflammation, heart diseases, coagulation, rheumatism, hypertension, Alzheimer’s disease	Increases insulin secretion and insulin sensitivity, improves glucose tolerance	[84,85,86]
18. *Artemisia absinthium*	Rhizome	Diabetes, wounds, indigestion, gastritis, anemia, hepatitis, cardiovascular diseases, gall bladder disorders	Increases insulin sensitivity, improves glucose uptake, enhances GLUT-4 translocation	[87,88,89]
19. *Artocarpus heterophyllus*	Leaves, rhizome	Diabetes, diarrhea, malaria, wounds, anemia, inflammation	Lowers blood glucose levels, decreases glycosylated hemoglobin levels	[78,90]
20. *Asparagus racemosus*	Roots	Diabetes, constipation, ulcers, stomach disorders, cough, inflammation	Increases insulin secretion and action, improves β-cell function, inhibits carbohydrate digestion and absorption	[91,92,93,94]
21. *Atractylodes japonica*	Rhizome	Diabetes, rheumatism, gastrointestinal diseases, influenza, night blindness, diuretic, stomachic	Lowers blood glucose levels, reduces insulin resistance, improves glucose uptake	[95,96]
22. *Azadirachta indica*	Leaves	Diabetes, malaria skin diseases, infections, cardiovascular diseases, intestinal worms	Lowers blood glucose levels, increases insulin secretion, improves pancreatic β-cell function, inhibits α-amylase and α-glucosidase activity, enhances glucose uptake	[56,97,98]
23. *Balanites aegyptiaca*	Fruit	Diabetes, wounds, asthma, malaria, diarrhea, hemorrhoids, fever, infections	Increases insulin secretion, improves glucose uptake, inhibits α-glucosidase activity	[99,100]
24. *Berberis vulgaris*	Root, bark	Diabetes, eye infections, piles, wounds, snake bites, hemorrhoids, dysentery	Reduces blood glucose levels, increases insulin secretion	[101,102]
25. *Bidens pilosa*	Root	Diabetes, wounds, hepatitis, diarrhea, urinary tract infections, cold, glandular sclerosis	Increases plasma insulin, improves glucose tolerance, protects or prevents islet degeneration	[103,104]
26. *Bougainvillea spectabilis*	Flowers, leaves	Diabetes, inflammation, ulcers, sore throat, infections, contraceptive	Regenerates β-cell function, increases plasma insulin levels, reduces intestinal glucosidase activity	[105,106]
27. *Brassica juncea*	Leaves, seeds	Diabetes, arthritis, rheumatism, back pain, coughs, paralysis	Increases insulin secretion and glucose utilization	[16,107]
28. *Bridelia ferruginea*	Leaves, stem bark	Diabetes, headache, arthritis, fever, inflammation	Lowers blood glucose levels, inhibits α-amylase and α-glucosidase activity	[108,109]
29. *Bunium persicum*	Seeds	Diabetes, diarrhea, gastrointestinal disorders, inflammation, obesity, asthma	Lowers blood glucose levels, improves glucose uptake and utilization	[56,110,111]
30. *Caesalpinia decapetala*	Leaves	Diabetes, indigestion, flatulence, stomach aches, constipation, fever	Lowers blood glucose levels, protects pancreatic beta cells, decreases oxidative stress	[112,113]
31. *Calendula officinalis*	Leaves, bark	Diabetes, fever, infections, wounds, menstrual irregularity, poor eyesight, inflammation, ulcers	Lowers blood glucose levels, increases plasma insulin levels	[114,115]
32. *Camellia sinensis*	Leaves	Diabetes, heart diseases, diuretic, astringent, stimulant, flatulence	Increases insulin secretion and action, inhibit insulin glycation, DPP-IV enzyme, and α-amylase activity, improves glucose tolerance	[116,117]
33. *Capsicum frutescens*	Whole plant	Diabetes, gastrointestinal disorders, toothache, pain, muscle spasms, fever, infections	Increases insulin secretion and insulin sensitivity, improves glucose uptake	[118,119]
34. *Carica papaya*	Fruit, leaves	Diabetes, gastrointestinal disorders, dengue, malaria, nerve pains, insomnia, constipation	Lowers blood glucose levels, increases insulin secretion, suppresses glucagon secretion	[120,121]
35. *Cassia alata*	Leaves, seeds	Diabetes, skin diseases, rheumatism, constipation, ringworm, infections, inflammation	Lowers blood glucose levels, inhibits α-glucosidase activity	[122,123]
36. *Cassia fistula*	Stalk	Diabetes, wounds, constipation, piles, skin diseases, asthma, liver diseases, rheumatism, leprosy	Lowers blood glucose levels, increases insulin secretion, improves glucose uptake and utilization	[56,124,125,126,127]
37. *Catharanthus roseus*	Leaves, roots	Diabetes, hypertension, menstrual irregularity, cancer, wounds, muscle pain	Lowers blood glucose levels, increases insulin sensitivity, improves glucose uptake and utilization	[128,129,130]
38. *Cecropia obtusifolia*	Root bark	Diabetes, asthma, bronchitis, heart diseases, inflammation, wounds, hypertension	Lowers blood glucose levels, decreases glycosylated hemoglobin levels	[78,131]
39. *Cichorium intybus*	Bark, leaves	Diabetes, constipation, wounds, liver diseases	Increases insulin secretion and insulin sensitivity, improves glucose uptake	[78,132]
40. *Cinnamomum zeylanicum*	Bark	Diabetes, common cold, flu, gastrointestinal disorders, bacterial infections, headache, stomach pain	Increases plasma insulin levels, increases insulin sensitivity, inhibits α-amylase activity	[133,134]
41. *Citrus limon*	Fruit	Diabetes, hypertension, infections, scurvy, sore throat, rheumatism	Lowers plasma glucose levels, inhibits α-amylase activity	[135,136]
42. *Citrus x aurantium*	Fruit	Diabetes, insomnia, indigestion, constipation, heartburn, nausea, cardiovascular diseases	Lowers blood glucose levels, increases insulin secretion	[137,138]
43. *Cola nitida*	Seeds	Diabetes, dysentery, fatigue, CNS stimulant, morning sickness, migraine, indigestion, wounds	Lowers blood glucose levels, increases serum insulin levels	[139,140]
44. *Coptis chinensis*	Rhizome	Diabetes, sore throat, whooping cough, dysentery, neurodegenerative diseases	Lowers blood glucose levels, increases insulin sensitivity, improves glucose uptake	[141,142]
45. *Cornus officinalis*	Fruit, seeds	Diabetes, pain, inflammation, cardiovascular diseases, liver, and kidney diseases	Lowers blood glucose levels, increases insulin secretion, inhibits α-glucosidase activity, increases GLUT-4 expression	[143,144]
46. *Curcuma longa*	Rhizome	Diabetes, gastric, inflammation, infections, cough, pain, liver diseases	Lowers blood glucose levels, inhibits α-amylase and α-glucosidase activity, increases insulin secretion, improves peripheral glucose uptake, reduces insulin resistance	[78,145,146]
47. *Cudrania cochinchinensis*	Bark, roots	Diabetes, hepatitis, scabies, bruises, gonorrhea, jaundice, rheumatism	Lowers blood glucose levels, increases insulin secretion, improves glucose uptake and utilization, inhibits DPP-IV enzyme and α-glucosidase activity	[56,147,148]
48. *Cyamopsis tetragonoloba*	Fruit	Diabetes, night blindness, arthritis, sprains, constipation, asthma, liver diseases, obesity	Increases insulin secretion, protects pancreatic beta cells, decreases glycosylated hemoglobin levels	[149,150]
49. *Dalbergia sissoo*	Bark	Diabetes, stomach disorders, dysentery, skin diseases, syphilis, nausea, gonorrhea	Lowers blood glucose levels, reduces serum triglyceride and cholesterol levels	[56,151,152]
50. *Eriobotrya japonica*	Leaves, seeds	Diabetes, bronchitis, inflammation, cough	Lowers blood glucose levels, reduces insulin resistance, improves glucose tolerance	[153,154]
51. *Eucalyptus citriodora*	Leaves	Diabetes, fever, pain, sinusitis, bronchitis, asthma, chronic rhinitis,	Increases insulin secretion, improves glucose uptake, inhibits insulin glycation and DPP-IV enzyme activity, decreases starch digestion	[155,156]
52. *Eucalyptus globulus*	Leaves	Diabetes, cough, cold, wounds, fungal infections, fever, sore throat, pain	Increases insulin secretion, improves glucose uptake	[157,158]
53. *Euclea undulata*	Root, bark	Diabetes, cough, chest pain, diarrhea, headache, toothache	Lowers blood glucose levels, inhibits α-glucosidase activity	[78,159]
54. *Eugenia jambolana*	Seeds	Diabetes, skin ulcers, gastritis, constipation, sore throat, liver, and kidney diseases	Lowers blood glucose levels, improves pancreatic β-cell function, increases insulin secretion, inhibits sucrase and maltase activity, improves glucose uptake and metabolism	[56,160,161]
55. *Euphorbia hirta*	Leaves	Diabetes, respiratory diseases, diarrhea, jaundice, tumors, gonorrhea	Increases insulin release from beta cells, inhibits α-glucosidase activity	[162,163]
56. *Ficus benghalensis*	Bark, leaves	Diabetes, hypertension, dysentery, diarrhea, pain, ulcers, asthma	Decrease carbohydrate digestion and absorption, lowers blood glucose levels	[164,165]
57. *Garcinia kola*	Seeds	Diabetes, diarrhea, food poisoning, bacterial infections, cough, liver diseases	Inhibits α-amylase activity, decreases glycosylated hemoglobin levels	[166,167]
58. *Glycine max*	Seeds	Diabetes, cardiovascular diseases, obesity, cancer	Reduces insulin resistance, improves glucose tolerance	[168,169]
59. *Glycyrrhiza glabra*	Roots	Diabetes, epilepsy, respiratory diseases, paralysis, jaundice, rheumatism	Lowers blood glucose levels, increases insulin secretion	[56,170]
60. *Gymnema sylvestre*	Leaves	Diabetes, asthma, bronchitis, constipation, jaundice, dyspepsia, hemorrhoids, obesity	Lowers blood glucose levels, regenerates beta cells, increases insulin secretion, improves glucose tolerance	[171,172]
61. *Harungana madagascariensis*	Leaves	Diabetes, cancer, hernia, hypertension, jaundice, malaria, yellow fever	Lowers blood glucose levels, inhibits α-amylase activity	[16,173]
62. *Helicteres isora*	Roots	Diabetes, diarrhea, snake bites, gastrointestinal disorders, spasms	Lowers blood glucose levels, improves glucose uptake	[174,175]
63. *Heritiera fomes*	Bark	Diabetes, diarrhea, constipation, dysentery, dermatitis, scabies, goiter	Decreases carbohydrate digestion and glucose absorption, lowers blood glucose levels, increases insulin secretion, improves glucose tolerance, inhibits DPP-IV enzyme activity	[26,51,176]
64. *Hibiscus esculentus*	Roots, seeds	Diabetes, gastric irritations, inflammatory diseases, wounds, and boils	Lowers blood glucose levels, improves β-cell function, increases insulin secretion	[177,178]
65. *Hibiscus rosa-sinensis*	Leaves	Diabetes, cough, diarrhea, dysentery, pain, contraceptive	Reduces glucose absorption, lowers blood glucose levels, increases insulin secretion and hepatic glucose utilization, improves glucose tolerance, inhibits DPP-IV activity	[179,180]
66. *Jatropha curcas*	Leaves	Diabetes, fever, bacterial and fungal infections, jaundice, muscle pain	Lowers fasting blood glucose levels, improves glucose uptake and utilization	[181,182]
67. *Lantana camara*	Leaves	Diabetes, asthma, malaria, chicken pox, hypertension, measles	Lowers elevated blood glucose levels, improves glucose tolerance	[183,184]
68. *Linum usitatissimum*	Seeds	Diabetes, diarrhea, gastrointestinal infections, asthma, bronchitis, atherosclerosis	Lowers blood glucose levels, increases insulin secretion, improves glucose uptake and metabolism	[56,185]
69. *Mangifera indica*	Leaves, seeds	Diabetes, constipation, piles, dysentery, asthma, anemia, hypertension, hemorrhage,	Lowers blood glucose levels, increases insulin secretion, improves glucose uptake, inhibits α-glucosidase and DPP-IV activity	[56,186,187]
70. *Momordica charantia*	Leaves, seeds	Diabetes, malaria, hypertension, scabies, liver diseases, obesity, ulcers, measles	Lowers blood glucose levels, increases insulin secretion and glucose uptake, improves glucose tolerance, decreases gluconeogenesis, inhibits α-glucosidase activity	[56,134,188]
71. *Moringa oleifera*	Leaves	Diabetes, asthma, enlarged liver, bacterial infections, eye problems, piles, influenza, diuretic	Reduces glucose absorption, lowers blood glucose levels, improves glucose uptake, inhibits α-amylase activity	[189,190]
72. *Murraya koenigii*	Leaves	Diabetes, piles, dysentery, itching, bruises, inflammation	Lowers blood glucose levels, inhibits α-amylase and α-glucosidase activity	[78,191]
73. *Musa sapientum*	Flowers	Diabetes, dysentery, ulcers, hypertension, pain, inflammation, snake bites	Lowers blood glucose levels, increases insulin secretion, decreases glucosylated hemoglobin levels	[192,193]
74. *Nigella sativa*	Seeds	Diabetes, hypertension, gastrointestinal disorders, back pain, paralysis, heart diseases, bacterial infections, malaria	Decreases carbohydrate digestion and absorption, lowers blood glucose levels, increases insulin secretion and sensitivity, improves glucose uptake and utilization	[194,195]
75. *Ocimum basicllicum*	Leaves	Diabetes, headaches, constipation, coughs, kidney diseases, warts	Inhibits α-amylase and α-glucosidase activity, reduces oxidative stress, inhibits glycogenolysis	[196,197,198]
76. *Ocimum sanctum*	Leaves	Diabetes, ringworm, skin diseases, dysentery, dyspepsia, bronchitis, asthma	Increases insulin secretion, improves glucose uptake and utilization	[149,199]
77. *Olea europaea*	Leaves	Diabetes, constipation, urinary tract infections, asthma, hypertension, intestinal diseases	Lowers blood glucose levels, increases antioxidant activity	[200,201]
78. *Panax ginseng*	Roots	Diabetes, insomnia, anorexia, confusion, hemorrhage	Improves peripheral insulin action, increases insulin sensitivity, decreases carbohydrate absorption	[202,203]
79. *Panda oleosa*	Stem bark	Diabetes, HIV/AIDS, wounds, rheumatism, intestinal parasites	Lowers blood glucose levels, improves glucose tolerance	[16,204]
80. *Phaseolus vulgaris*	Seeds	Diabetes, hypertension, obesity, blood cancer	Reduces insulin resistance, inhibits α-amylase and DPP-IV enzyme activity	[149,205]
81. *Phyllanthus amarus*	Leaves	Diabetes, spleen, liver and kidney diseases, gonorrhea, stomach problems	Lowers blood glucose levels, increases insulin secretion, improves insulin sensitivity	[206,207]
82. *Plantago ovata*	Husk	Diabetes, constipation, diarrhea, hypercholesterolemia, hypertension, hemorrhoids	Improves glucose tolerance, decreases carbohydrate digestion and glucose absorption	[208,209]
83. *Pterocarpus marsupium*	Bark	Diabetes, dysentery, cough, diarrhea, skin diseases, wounds, ulcer	Improves pancreatic β-cell function, increases insulin secretion, improves glucose uptake	[149,210,211]
84. *Punica granatum*	Flowers	Diabetes, urinary tract infections, arthritis, sore throat, skin diseases, anemia	Improves β-cell function, increases insulin secretion	[210,212,213]
85. *Rehmannia glutinosa*	Roots	Diabetes, anemia, obesity, kidney diseases, osteoporosis	Improves pancreatic β-cell function, increases insulin secretion, improves glucose uptake, decreases oxidative stress	[214,215]
86. *Santalum album*	Bark	Diabetes, jaundice, diarrhea, dysentery, liver tonic, inflammation, hypertension	Lowers blood glucose levels, increases insulin secretion, improves glucose uptake and utilization	[56,216]
87. *Selaginella bryopteris*	Leaves	Diabetes, fever, epilepsy, constipation, colitis, cancer, urinary tract infections	Lowers blood glucose levels, increases insulin secretion, improves glucose uptake and utilization	[56,217]
88. *Sesamum indicum*	Seeds	Diabetes, constipation, hypertension, high cholesterol, athlete’s foot	Inhibits α-amylase and α-glucosidase activity, exerts antioxidant activity	[56,218,219]
89. *Solanum nigrum*	Leaves	Diabetes, pneumonia, toothache, stomach ache, fever, tumor, tonsilitis	Lowers blood glucose levels, increases insulin secretion, decreases gluconeogenesis, increases glycogenesis	[220,221]
90. *Spirulina platensis*	Whole plant	Diabetes, hypercholesterolemia, atherosclerosis, obesity	Lowers blood glucose levels, increases insulin secretion, improves glucose tolerance, inhibits DPP-IV activity	[222,223]
91. *Swertia chirayita*	Bark, leaves	Diabetes, malaria, hypertension, epilepsy, liver diseases, weight loss	Lowers blood glucose levels, increases insulin secretion, improves glucose uptake and metabolism, inhibits α-amylase and α-glucosidase	[56,224]
92. *Tamarindus indica*	Seeds	Diabetes, diarrhea, dysentery, constipation, abdominal pain, wounds, malaria	Lowers blood glucose levels, increases insulin secretion	[56,225]
93. *Terminalia arjuna*	Bark	Diabetes, cardiotonic, anemia, viral infections, venereal diseases, ulcers	Lowers blood glucose levels, increases insulin secretion, improves glucose uptake and utilization	[56,226]
94. *Terminalia chebula*	Fruit	Diabetes, fever, astringent, constipation, dementia	Improves β-cell function, increases insulin secretion, reduces glycosylated hemoglobin levels	[227,228]
95. *Tinospora cordifolia*	Leaves, roots, stem	Diabetes, dysentery, diarrhea, snake bites, asthma, fever, jaundice	Increases insulin secretion, inhibits gluconeogenesis, increases insulin sensitivity	[149,229]
96. *Trigonella foenum-graecum*	Seeds	Diabetes, bronchitis, pneumonia, indigestion, dysentery, high cholesterol	Lowers blood glucose levels, increases insulin secretion, improves glucose uptake and utilization	[56,134,230,231]
97. *Urtica dioica*	Leaves	Diabetes, cardiovascular diseases, anemia, rhinitis, arthritis, gout, wounds	Increases insulin sensitivity, improves glucose tolerance	[232,233]
98. *Vernonia amygdalina*	Leaves	Diabetes, gastrointestinal disorders, amoebic dysentery, malaria, helminth infections	Lowers elevated blood glucose levels, inhibits gluconeogenesis and glycogenolysis	[234,235]
99. *Withania coagulans*	Fruit	Diabetes, insomnia, impotence, nervous exhaustion, asthma, liver diseases	Lowers blood glucose levels, improves glucose tolerance	[56,236]
100. *Zingiber officinale*	Rhizome	Diabetes, nausea, high cholesterol, heartburn, indigestion, diarrhea, asthma	Lowers fasting blood glucose levels, increases insulin secretion	[119,237]

**Table 2:** Phytoconstituents of antidiabetic medicinal plants and their pharmacological effects.

Medicinal Plants	Parts	Phytoconstituents	Pharmacological Effects	References
*Abrus precatorius*	Leaves, seeds	Luteolin, lupenone, 24-methylene cycloartenol	Maintains blood glucose levels, promotes insulin secretion, prevents oxidative stress, inhibits inflammation in pancreatic tissues	[16,242,243]
2. *Acacia arabica*	Bark, roots	Quercetin, kaempferol, catechin	Lowers blood glucose levels, increases insulin secretion, reduces insulin resistance, improves glucose tolerance, reduces oxidative stress	[24,244]
3. *Acacia catechu*	Bark	Catechin, epicatechin, catechu tannic acid, gallocatechin, kaempferol	Lowers blood glucose levels, increases plasma insulin levels, reduces insulin resistance, and improves glucose uptake, inhibits α-amylase and α-glucosidase activity	[24,244,245,246,247]
4. *Aegle marmelos*	Leaves	Rutin, β-sitosterol, aegelinosides A and B, aegeline, marmelosin	Lowers plasma glucose levels, reduces insulin resistance, decreases glycosylated hemoglobin levels, inhibits α-glucosidase activity, improves β-cell function	[248,249,250,251,252]
5. *Aframomum melegueta*	Fruit, leaves	6-paradol, 6-shogaol, 6-gingerol, oleanolic acid	Decreases blood glucose and cholesterol levels, improve glucose tolerance and utilization, inhibits lipid synthesis by adipocytes	[16,253,254,255]
6. *Ageratum conyzoides*	Leaves	Kaempferol, precocene II	Lowers blood glucose levels, increases plasma insulin levels, improves glucose uptake	[16,256]
7. *Albizia lebbeck*	Bark, pods	Lupeol, oleanolic acid, docosanoic acid, β-sitosterol, catechin, friedelin	Decreases blood glucose and glycosylated hemoglobin levels, reduces nitric oxide, increases insulin levels, activates GLUT2 and GLUT4	[244,250,255,257,258,259]
8. *Albizia adianthifolia*	Bark, leaves	β-caryophyllene, viridiflorol	Lowers blood glucose levels, increases insulin secretion and sensitivity, reduces glucose absorption, triglyceride, and cholesterol levels	[67,260]
9. *Allium cepa*	Bulb	Alliin, quercetin, S-methyl cysteine sulfoxide	Reduces fasting glucose levels, increases insulin secretion and sensitivity, decreases triglyceride levels	[16,261,262]
10. *Allium sativum*	Bulb	Allicin, alliin, diallyl disulfide, quercetin, allyl propyl disulfide	Lowers blood glucose levels, increases insulin secretion and sensitivity, decreases cholesterol and triglyceride levels	[71,261,262,263]
11. *Aloe vera*	Leaves	Lophenol, aloin, aloetic acid, emodin, glucomannan	Lowers blood glucose levels, increases insulin secretion, improves glucose tolerance, prevents oxidative stress	[16,264,265,266]
12. *Anacradium occidentale*	Leaves, stem bark	Anacardic acid, lectin	Delays glucose absorption, reduces oxidative stress, inhibits α-glucosidase activity	[16,267]
13. *Anemarrhena asphodeloides*	Rhizome	Mangiferin, neomangiferin, sarsasapogenin	Reduces fasting blood glucose levels, improves glucose tolerance, reduces cholesterol and triglyceride levels, improves diabetic complications	[78,268,269,270]
14. *Annona salzmannii*	Leaves, bark	α-copaene, β-caryophyllene, δ-cadinene	Lowers blood glucose levels, increases insulin secretion, improves glucose uptake, reduces glucose absorption, cholesterol, and triglyceride levels	[80,260]
15. *Annona squamosa*	Leaves	Rutin, quercetin, isoquercetin	Lowers blood glucose levels, increases insulin secretion, improves glucose tolerance, reduces glycosylated hemoglobin levels	[10,249,262,271]
16. *Anogeissus latifolia*	Bark	Ellagic acid, β-sitosterol, 3,4,3-tri-*O*-methylellagic acid	Lowers plasma glucose and glycosylated hemoglobin levels, increases insulin levels, improves β-cell function	[250,272,273]
17. *Arachis hypogaea*	Seeds	Resveratrol, catechin, rutin, quercetin	Lowers blood glucose levels, increases insulin secretion and glucose uptake, reduces oxidative stress, inhibits α-amylase and α-glucosidase activity	[244,249,262,274]
18. *Artemisia absinthium*	Rhizome	α and β thujones, thujyl alcohol, azulene, cadinene	Lowers blood glucose levels, activates adenosine monophosphate-activated protein kinase, increases insulin sensitivity	[16,275,276]
19. *Artocarpus heterophyllus*	Leaves, rhizome	Chrysin, silymarin, isoquercetin	Lowers blood glucose levels, improves β-cell function and glucose tolerance, increases insulin sensitivity, inhibits Pro-inflammatory cytokines	[78,271,277,278]
20. *Asparagus racemosus*	Roots	Asparagamine, asparagine, kaempferol, quercetin	Lowers blood glucose levels, increases insulin secretion, improves glucose uptake and tolerance	[93,256,262]
21. *Atractylodes japonica*	Rhizome	Atractans A, B, C, atractylenolide III	Lowers blood glucose levels, decreases insulin resistance	[95,96,279]
22. *Azadirachta indica*	Leaves	Azadirachtin, nimbin, rutin, quercetin, campestrol	Lowers blood glucose levels, improves β-cell function, increases insulin secretion, reduces cholesterol and triglyceride levels	[97,98,249,280]
23. *Balanites aegyptiaca*	Fruit, seeds	Balantin 1, 2, diosgenin, 3,4,6-tri-*O*-methyl-D-glucose, triethylphosphine	Increases serum insulin and c-peptide levels, increases glucose metabolism, decreases gluconeogenesis	[16,281]
24. *Berberis vulgaris*	Root bark	Berberine, berbamine	Increases insulin secretion, improves insulin sensitivity, inhibits α-glucosidase and aldose reductase activity	[102,282,283]
25. *Bidens pilosa*	Roots	Cytopiloyne, apigenin, luteolin, kaempferol, quercetin	Lowers blood glucose and glycosylated hemoglobin levels, increases insulin expression and secretion from beta cells, stimulates glucose metabolism, increases insulin sensitivity to cells	[16,242,284,285,286]
26. *Bougainvillea spectabilis*	Flowers, leaves	Pinitol, quercetin, β-sitosterol	Lowers fasting blood glucose and glycosylated hemoglobin levels, increases insulin secretion, improves insulin sensitivity	[16,250,262,287]
27. *Brassica juncea*	Leaves, seeds	Cinnamic acid, kaempferol, aniline	Lowers blood glucose levels, increases insulin secretion and glucose uptake, improves glucose tolerance	[16,256,288]
28. *Bridelia ferruginea*	Leaves, stem bark	Epigallocatechin, epigallocatechin gallate	Lowers blood glucose levels, improves glucose tolerance, enhances insulin secretion, decreases gluconeogenesis	[16,289,290]
29. *Bunium persicum*	Seeds	Linoleic acid, palmitic acid, kaempferol, camphene, linalool	Lowers blood glucose levels, increases insulin levels in blood, improves insulin sensitivity, enhances glucose uptake and tolerance	[256,291,292,293,294]
30. *Caesalpinia decapetala*	Leaves	Quercitrin, kaempferol, astragalin, apigenin-7-rhamnoside	Decreases fasting blood glucose levels, increases insulin levels in blood, enhances antioxidant activity, improves glucose uptake, decreases nitric oxide	[16,256,295,296]
31. *Calendula officinalis*	Leaves, bark	Caffeic acid, quercetin, esculetin	Lowers blood glucose and glycosylated hemoglobin levels, increases insulin secretion, reduces diabetic oxidative stress, increases GLUT4 expression in adipocytes, improves glucose utilization	[16,262,297,298]
32. *Camellia sinensis*	Leaves	Rutin, quercitrin	Lowers blood glucose levels, improves β-cell function, increases insulin secretion, improves glucose tolerance	[117,249,295]
33. *Capsicum frutescens*	Whole plant	Capsaicin, β-carotene	Lowers blood glucose levels, increases insulin levels, improves glucose tolerance, inhibits pro-inflammatory cytokines	[119,299,300]
34. *Carica papaya*	Fruit, leaves	Chlorogenic acid, coumarin compounds	Lowers blood glucose levels, stimulates insulin secretion, increases insulin sensitivity, inhibits α-amylase, α-glucosidase, glucose-6-phosphatase, and aldose reductase activity	[16,301,302]
35. *Cassia alata*	Leaves, seeds	Emodin, kaempferol, β-sitosterol	Lowers blood glucose levels, increases insulin secretion, enhances insulin sensitivity, inhibits phosphoenolpyruvate, carboxykinase, glucose-6-phosphatase activity	[16,250,256,266]
36. *Cassia fistula*	Stalk	Lupeol, kaempferol, catechin, epicatechin	Lowers blood glucose and glycosylated hemoglobin levels, increases insulin levels, reduces nitric oxide, improves glucose tolerance	[244,246,257,303]
37. *Catharanthus roseus*	Leaves, roots	Gallic acid, chlorogenic acid, vindoline I	Lowers blood glucose levels, stimulates insulin secretion, improves glucose tolerance, decreases pro-inflammatory cytokines	[16,301,304,305]
38. *Cecropia obtusifolia*	Root, bark	Isoorientin, stigmast-4-en-3-one, chlorogenic acid, β-sitosterol	Reduces blood glucose levels, improves insulin sensitivity, enhances glucose uptake, decreases cholesterol and triglyceride levels, inhibits glucose-6-phosphatase and hepatic glucose, improves glucose tolerance	[78,306,307]
39. *Cichorium intybus*	Bark, leaves	Chlorogenic acid, chicoric acid, gallic acid, kaempferol, quercetin, β-sitosterol	Lowers blood glucose levels, stimulates insulin release, improves insulin sensitivity, inhibits α-amylase, α-glucosidase, glucose-6-phosphatase activity, prevents oxidative stress	[22,78,132,301,308]
40. *Cinnamomum zeylanicum*	Bark	Cinnamaldehyde, eugenol	Decreases blood glucose levels, reduces insulin resistance, inhibits α-glucosidase activity and formation of advanced glycated end products, inhibits sugar binding to albumin	[134,309,310]
41. *Citrus limon*	Fruit	Diosmin, hesperetin	Lowers blood glucose levels, increases insulin secretion, enhances glucose utilization, stimulates β-endorphine secretion from adrenal glands, inhibits gluconeogenesis	[16,311,312]
42. *Citrus x aurantium*	Fruit	Naringin, naringenin, epigallocatechin-3-gallate	Decreases blood glucose levels, increases insulin secretion, improves glucose tolerance, increases GLUT4 translocation in skeletal muscles, decreases gluconeogenesis	[16,289,290,313]
43. *Cola nitida*	Seeds	D-catechin, L-epicatechin, naringenin, apigenin	Lowers blood glucose levels, increases insulin sensitivity, decreases oxidative stress, inhibits α-amylase and α-glucosidase activity	[16,244,246]
44. *Coptis chinensis*	Rhizome	Berberine, jatrorrhizine	Lowers blood glucose levels, enhances aerobic glycolysis, inhibits gluconeogenesis, increases insulin secretion and insulin sensitivity	[33,282,314]
45. *Cornus officinalis*	Fruit, seeds	Gymnemagenin, gymnemic acid, ursolic acid	Lowers fasting blood glucose levels, increases insulin secretion, improves glucose uptake and tolerance, inhibits protein glycation	[143,279,315,316]
46. *Curcuma longa*	Rhizome	Curcumin, turmerin	Decreases fasting blood glucose, glycosylated hemoglobin, triglyceride, and cholesterol levels, inhibits α-amylase, α-glucosidase activity, and diabetic inflammatory processes	[78,317,318]
47. *Cudrania cochinchinensis*	Bark, roots	Kaempferol, vanillin, β-sitosterol	Lowers blood glucose levels, increases insulin levels, decreases serum advanced glycation end products, improves glucose uptake, reduces insulin resistance	[250,256,319,320]
48. *Cyamopsis tetragonoloba*	Fruit	Quercetin, kaempferol, gallic acid	Lowers plasma glucose levels, increases insulin secretion, improves glucose tolerance, decreases triglyceride levels	[16,256,262,304]
49. *Dalbergia sissoo*	Bark	Biochanin A, tectorigenin, rhamnoglucoside, dalbergin, dalbergichromene	Lowers blood glucose levels, improves insulin sensitivity and glucose tolerance, reduces insulin resistance	[321,322,323]
50. *Eriobotrya japonica*	Leaves, seeds	Cinchonain-Ib, timosaponin, chlorogenic acid, epicatechin	Lowers blood glucose, total cholesterol, and triglyceride levels, enhances insulin secretion and sensitivity, improves glucose tolerance	[246,279,301,324,325]
51. *Eucalyptus citriodora*	Leaves	Betulinic acid, gallic acid, quercitrin, isoquercitrin, rhodomyrtosone E	Lowers blood glucose levels, increases insulin secretion and sensitivity, improves glucose tolerance and antioxidant activity, decreases triglyceride levels,	[155,295,304,326]
52. *Eucalyptus globulus*	Leaves	Eucalyptol, rutin, sesquiterpene	Lowers blood glucose levels, improves β-cell function, increases insulin secretion, reduces oxidative stress	[157,249,327]
53. *Euclea undulata*	Rootbark	Botulin, lupeol, epicatechin	Decreases serum glucose, increases insulin levels, improves insulin sensitivity, decreases glycosylated hemoglobin levels	[78,246,257]
54. *Eugenia jambolana*	Seeds	Ellagic acid, gallic acid, chlorogenic acid	Lowers blood glucose levels, increases insulin sensitivity, improves β-cell function, improves glucose tolerance, inhibits α-amylase, α-glucosidase, and glucose-6-phosphatase activity	[11,272,301,304]
55. *Euphorbia hirta*	Leaves	Quercetin, kaempferol, gallic acid	Lowers blood glucose levels, increases insulin secretion, improves glucose tolerance, decreases triglyceride levels, enhances glucose uptake	[162,256,262,304]
56. *Ficus benghalensis*	Bark, leaves	Rutin, gallic acid, leucopelargonidin-3-*O*-α-rhamnopyranoside, lupeol, α-amyrin acetate	Decreases blood glucose levels, improve glucose tolerance and β-cell function, increases insulin secretion,	[249,328,329,330]
57. *Garcinia kola*	Seeds	Kolaviron, ascorbic acid	Decreases blood glucose level, stimulates insulin secretion, improves glucose utilization, inhibits glucose-6-phosphatase, exhibits free radical scavenging activity	[16,331,332]
58. *Glycine max*	Seeds	Kaempferol, soyasaponin, genistein, β-sitosterol	Lowers blood glucose and glycosylated hemoglobin levels, increases insulin levels in blood, decreases insulin resistance, improves glucose uptake, inhibits glucose absorption	[16,250,256]
59. *Glycyrrhiza glabra*	Roots	Glycyrrhizin, glycyrrhetinic acid, isoliquiritin	Lowers postprandial rise in blood glucose levels, decreases glycosylated hemoglobin levels	[333,334,335]
60. *Gymnema sylvestre*	Leaves	Gymnemoside A,B,C,D,E,F, quercitol, lupeol, gymnemic acid	Lowers blood glucose and glycosylated hemoglobin levels, increases insulin secretion, inhibits glucose absorption in the small intestine	[149,257,315,336]
61. *Harungana madagascariensis*	Leaves	Harunganin, lupeol, betulinic acid, quercetin, β-sitosterol	Lowers blood glucose and glycosylated hemoglobin levels, increases insulin secretion, decreases insulin resistance, prevents diabetic nephropathy	[16,250,257,262,337,338]
62. *Helicteres isora*	Roots	Gallic acid, vanillin, *p*-coumaric acid	Lowers blood glucose levels, increases insulin levels in blood, decreases triglyceride levels, reduces serum advanced glycation end products concentration, improves glucose tolerance	[175,304,319,339]
63. *Heritiera fomes*	Bark	Stigmasterol, β-sitosterol, epicatechin, procyanidins, proanthocyanidins, quercitrin	Decreases blood glucose and glycosylated hemoglobin levels, increases insulin levels, reduces insulin resistance, improves glucose uptake	[26,176,250,340]
64. *Hibiscus esculentus*	Roots, seeds	Isoquercitrin, quercetin-3-*O*-gentiobioside	Decreases serum glucose levels, increases insulin secretion, improves glucose tolerance	[16,341]
65. *Hibiscus rosa-sinensis*	Leaves	Quercetin, cyanidin, thiamine, ascorbic acid, niacin	Decreases blood glucose concentration, increases insulin synthesis and secretion, reduces oxidative stress, improves endothelial functions, and reduces complications of type 2 diabetes mellitus	[179,262,342,343]
66. *Jatropha curcas*	Leaves	Rhoifolin, isoorientin, isoquercitrin	Decreases plasma glucose, cholesterol, and triglyceride levels, stimulates glucose uptake, inhibits DPP-IV activity	[241,306]
67. *Lantana camara*	Leaves	Lantanoside, ferulic acid, oleanolic acid, caffeic acid	Lowers blood glucose levels, increases insulin secretion, improves glucose utilization, reduces oxidative stress	[255,297,344]
68. *Linum usitatissimum*	Seeds	Caffeic acid, *p*-coumaric acid, ferulic acid	Lowers blood glucose and glycosylated hemoglobin levels, increases insulin secretion, reduces diabetic oxidative stress, enhances antioxidant activity	[297,339,344,345]
69. *Mangifera indica*	Leaves, seeds	Mangiferin, gallic acid, kaempferol, curcumin	Lowers fasting blood glucose levels, improves glucose tolerance, increases insulin secretion, reduces triglyceride and cholesterol levels, inhibits oxidative stress and diabetic inflammatory processes	[16,256,269,304,317]
70. *Momordica charantia*	Leaves, seeds	Charantin, vicine, momordicine II, oleanolic acid	Lowers blood glucose levels, stimulates insulin release, inhibits glucose-6-phosphatase and glucose transport in intestines	[22,134,255,336]
71. *Moringa oleifera*	Leaves	Quercetin, kaempferol, vanillin, chlorogenic acid	Lowers plasma glucose levels, increases insulin secretion, improves glucose tolerance, decreases the concentration of serum advanced glycation end products	[16,22,189,256,319]
72. *Murraya koenigii*	Leaves	Mahanimbine, isomahanine, ascorbic acid, kaempferol, quercetin	Lowers blood glucose levels, reduces triglyceride levels, inhibits α-amylase and α-glucosidase activity, increases insulin secretion, improves glucose tolerance	[78,191,346]
73. *Musa sapientum*	Flowers	Rutin, delphinidin, syringin	Lowers blood glucose levels, increases insulin secretion, reduces reactive oxygen species generation, prevents high glucose-induced cell proliferation	[16,249,347]
74. *Nigella sativa*	Seeds	Thymoquinone, thymol, α-pinene, oleic acid, linoleic acid	Lowers blood glucose, glycosylated hemoglobin, total cholesterol, and triglyceride levels, promotes insulin secretion, reduces insulin resistance, decreases oxidative stress	[291,348,349,350]
75. *Ocimum basicllicum*	Leaves	Linalool, linolen, eugenol, geraniol	Lowers blood glucose levels, improves glucose uptake, inhibits advanced glycation end products generation and α-glucosidase activity	[196,197,310,351]
76. *Ocimum sanctum*	Leaves	Eugenol, carvacrol, β-sitosterol, linalool	Lowers blood glucose levels, increases insulin secretion, decreases carbohydrate digestion and absorption, inhibits α-glucosidase activity, reduces insulin resistance	[149,248,250,310]
77. *Olea europaea*	Leaves	Oleuropein, oleanolic acid, luteolin	Maintains blood glucose levels, promotes insulin secretion, improves insulin sensitivity, reduces oxidative stress, inhibits gluconeogenesis	[16,242,255,352]
78. *Panax ginseng*	Roots	Ginsenoside Rb2, Rg2	Regenerates pancreatic beta cells, increases glucose uptake, reduces insulin resistance, and improves insulin sensitivity	[248,279,353]
79. *Panda oleosa*	Stem bark	Ginsenoside Rb2, protapananadiol/triol	Increases glucose uptake, reduces insulin resistance, and improves insulin sensitivity	[204,353]
80. *Phaseolus vulgaris*	Seeds	Hydroxycinnamic acid, rutin, quercetin, orientin, petunidin, catechin	Lowers blood glucose and glycosylated hemoglobin levels, increases insulin secretion, improves glucose tolerance, reduces oxidative stress	[16,149,244,249,262]
81. *Phyllanthus amarus*	Leaves	Oleanolic acid, ursolic acid	Lowers blood glucose levels, increases insulin secretion, improves glucose tolerance, inhibits oxidative stress-induced hepatic insulin resistance, inhibits gluconeogenesis	[16,255,316]
82. *Plantago ovata*	Husk	Kaempferol, catechin, myricetin, pinocembrin	Lowers blood glucose levels, increases insulin secretion, reduces insulin resistance, inhibits α-amylase and α-glucosidase activity	[208,244,256,354]
83. *Pterocarpus marsupium*	Bark	Epicatechin, marsupin, carsupin, marsupol	Lowers blood glucose levels, improves insulin sensitivity, enhances insulin release, improves glucose uptake	[149,246]
84. *Punica granatum*	Flowers	Gallic acid, rutin, nictoflorin	Lowers blood glucose levels, improves β-cell function, increases insulin secretion, improves glucose tolerance, decreases triglyceride levels	[16,249,304]
85. *Rehmannia glutinosa*	Roots	Catalpol, rehmannioside	Lowers blood glucose levels, prevents diabetic complications, promotes glucose utilization and glycogen synthesis, reduces oxidative stress	[214,279]
86. *Santalum album*	Bark	Spirosantalol, α-santalene, α-santalol, β-santalol, α-bergamotol	Lowers blood glucose and glycosylated hemoglobin levels, decreases total cholesterol and triglyceride levels	[355]
87. *Selaginella bryopteris*	Leaves	Gallic acid, rutin	Decreases plasma glucose and glycosylated hemoglobin levels, improves glucose tolerance, decreases triglyceride levels, inhibits inflammatory cytokines	[249,304,356]
88. *Sesamum indicum*	Seeds	Pinoresinol, sesamin, sesaminol	Lowers fasting blood glucose and glycosylated hemoglobin levels, inhibits α-glucosidase activity	[16,357,358]
89. *Solanum nigrum*	Leaves	Gallic acid, catechin, epicatechin, rutin, naringenin	Lowers blood glucose levels, improves β-cell function and glucose tolerance, increases insulin secretion, reduces insulin resistance, inhibits α-amylase and α-glucosidase activity	[220,244,246,249,304,313]
90. *Spirulina platensis*	Whole plant	*p*-coumaric acid, catechin, β-carotene	Lowers blood glucose levels, increases insulin levels, reduces insulin resistance, inhibits α-amylase and α-glucosidase activity, reduces oxidative stress and pro-inflammatory biomarkers	[222,244,300,339]
91. *Swertia chirayita*	Bark, leaves	Swerchirin, mangiferin, swertiamarin, amarogentin	Lowers blood glucose levels, promotes insulin release, inhibits glucosidase and glucuronidase activity	[30,268,269,336]
92. *Tamarindus indica*	Seeds	Apigenin, naringenin, catechin, epictaechin, taxifolin	Lowers blood glucose levels, increases insulin secretion, inhibits α-amylase and α-glucosidase activity, improves glucose tolerance, increases insulin sensitivity	[244,246,313,359]
93. *Terminalia arjuna*	Bark	Arjungenin, arjunolone, ellagic acid, derivatives of arjunic acid	Lowers blood glucose levels, increases insulin sensitivity, decreases free radical damage	[29,360]
94. *Terminalia chebula*	Fruit	Chebulagic acid, gallic acid, ellagic acid, tannic acid	Lowers blood glucose levels, improve glucose tolerance and lipid metabolism, stimulates glucose transport, decreases triglyceride levels	[245,304,360,361,362]
95. *Tinospora cordifolia*	Leaves, roots, stem	Tinosporaside, berberine, syringin	Lowers plasma glucose levels, stimulates insulin synthesis and secretion, decreases triglyceride levels, improves insulin sensitivity, inhibits gluconeogenesis	[149,282,363]
96. *Trigonella foenum-graecum*	Seeds	Galactomannan, diosgenin, coumarin	Decreases blood glucose levels, stimulates insulin release, inhibits α-glucosidase and aldose reductase activity, increases insulin sensitivity	[16,302,364,365]
97. *Urtica dioica*	Leaves	Quercetin, quercitrin, rutin	Lowers blood glucose and glycosylated hemoglobin levels, increases insulin secretion, reduces insulin resistance, improves antioxidant activity	[16,249,262,295]
98. *Vernonia amygdalina*	Leaves	Sobrerol, vernoamyoside E, luteolin, vitamin E	Lowers blood glucose and glycosylated hemoglobin levels, increases insulin secretion, enhances insulin sensitivity, reduces oxidative stress	[16,235,242,366,367]
99. *Withania coagulans*	Fruit	Withanolides, withacoagulin, withanosides, withaferin A	Lowers blood glucose levels, exhibits free radical scavenging activity, inhibits DPP-IV activity	[368,369]
100. *Zingiber officinale*	Rhizome	Gingerol, 6-paradol, 6-shogaol, camphene	Lowers blood glucose levels, increases insulin levels, improves glucose tolerance and utilization, decreases cholesterol levels	[16,253,254,293]

**Table 3:** Antidiabetic phytoconstituents of medicinal plants and their chemical structures.

Medicinal Plants	Phytoconstituents	Chemical Structure
*Abrus precatorius*	Lupenone	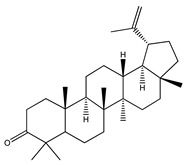
2. *Acacia arabica*	Quercetin	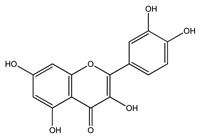
3. *Acacia catechu*	Gallocatechin	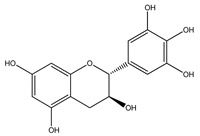
4. *Aegle marmelos*	Marmelosin	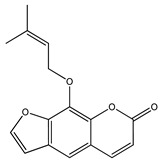
5. *Aframomum melegueta*	6-paradol	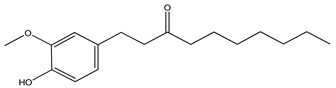
6. *Ageratum conyzoides*	Kaempferol	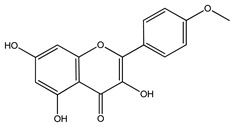
7. *Albizia lebbeck*	Friedelin	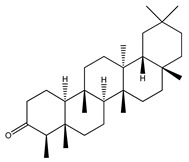
8. *Albizia adianthifolia*	Viridiflorol	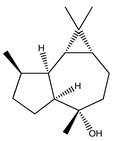
9. *Allium cepa*	Alliin	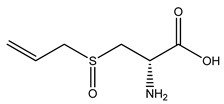
10. *Allium sativum*	Allicin	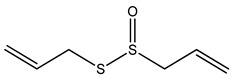
11. *Aloe vera*	Aloin	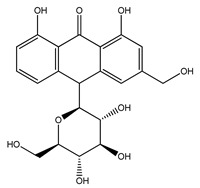
12. *Anacradium occidentale*	Anacardic acid	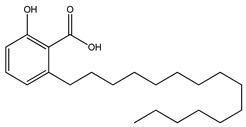
13. *Anemarrhena asphodeloides*	Sarsasapogenin	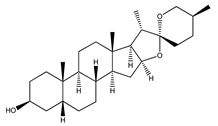
14. *Annona salzmannii*	β-caryophyllene	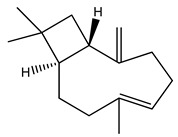
15. *Annona squamosa*	Rutin	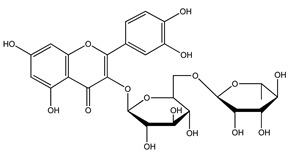
16. *Anogeissus latifolia*	β-sitosterol	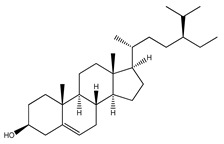
17. *Arachis hypogaea*	Resveratrol	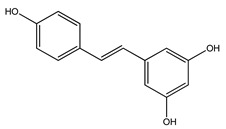
18. *Artemisia absinthium*	Azulene	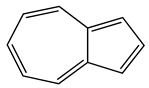
19. *Artocarpus heterophyllus*	Chrysin	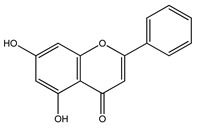
20. *Asparagus racemosus*	Asparagine	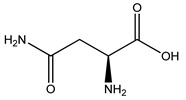
21. *Atractylodes japonica*	Atractylenolide III	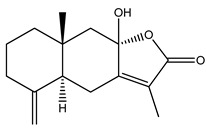
22. *Azadirachta indica*	Nimbin	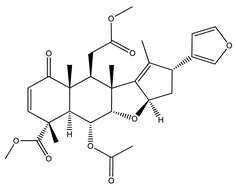
23. *Balanites aegyptiaca*	Diosgenin	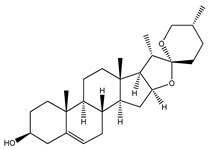
24. *Berberis vulgaris*	Berberine	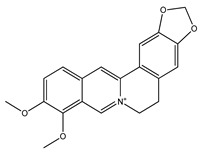
25. *Bidens pilosa*	Luteolin	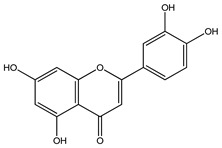
26. *Bougainvillea spectabilis*	Pinitol	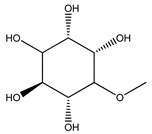
27. *Brassica juncea*	Cinnamic acid	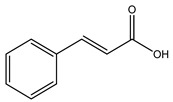
28. *Bridelia ferruginea*	Epigallocatechin gallate	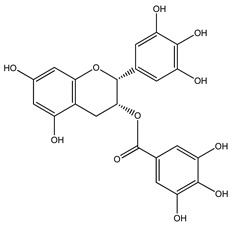
29. *Bunium persicum*	Palmitic acid	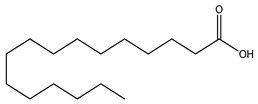
30. *Caesalpinia decapetala*	Astragalin	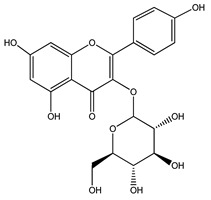
31. *Calendula officinalis*	Esculetin	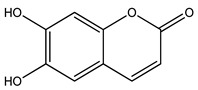
32. *Camellia sinensis*	Quercitrin	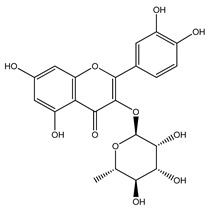
33. *Capsicum frutescens*	Capsaicin	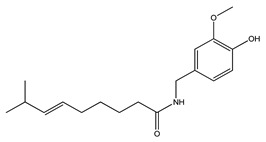
34. *Carica papaya*	Coumarin	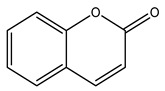
35. *Cassia alata*	Emodin	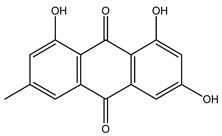
36. *Cassia fistula*	Lupeol	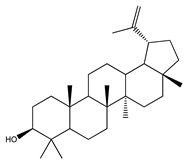
37. *Catharanthus roseus*	Vindoline	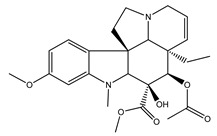
38. *Cecropia obtusifolia*	Isoorientin	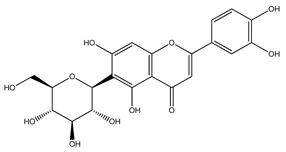
39. *Cichorium intybus*	Chlorogenic acid	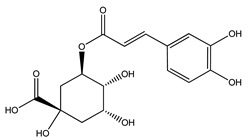
40. *Cinnamomum zeylanicum*	Cinnamaldehyde	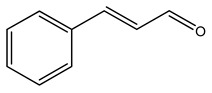
41. *Citrus limon*	Hesperetin	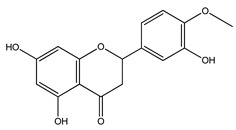
42. *Citrus x aurantium*	Naringin	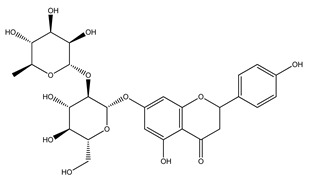
43. *Cola nitida*	Apigenin	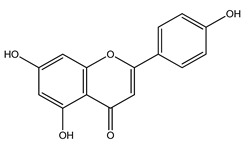
44. *Coptis chinensis*	Jatrorrhizine	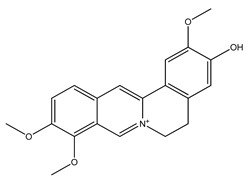
45. *Cornus officinalis*	Gymnemic acid	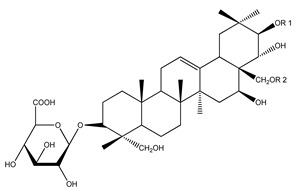
46. *Curcuma longa*	Curcumin	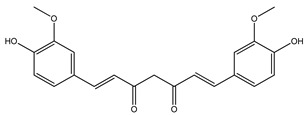
47. *Cudrania cochinchinensis*	Vanillin	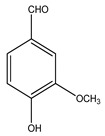
48. *Cyamopsis tetragonoloba*	Quercetin	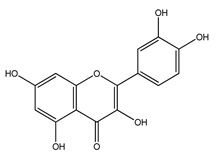
49. *Dalbergia sissoo*	Biochanin A	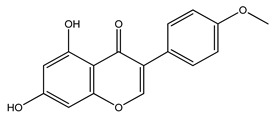
50. *Eriobotrya japonica*	Cinchonain ib	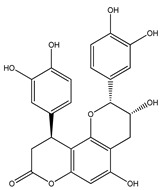
51. *Eucalyptus citriodora*	Rhodomyrtosone E	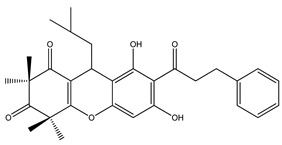
52. *Eucalyptus globulus*	Eucalyptol	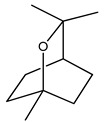
53. *Euclea undulata*	Epicatechin	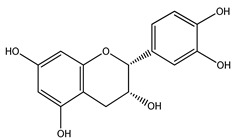
54. *Eugenia jambolana*	Ellagic acid	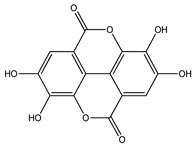
55. *Euphorbia hirta*	Gallic acid	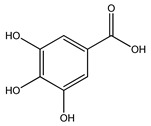
56. *Ficus benghalensis*	α-amyrin acetate	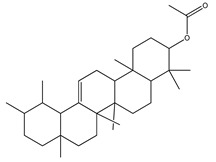
57. *Garcinia kola*	Kolaviron	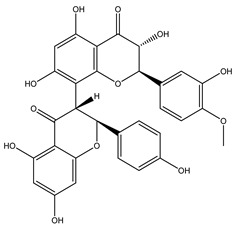
58. *Glycine max*	Genistein	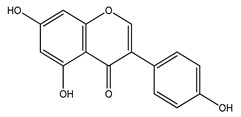
59. *Glycyrrhiza glabra*	Glycyrrhizin	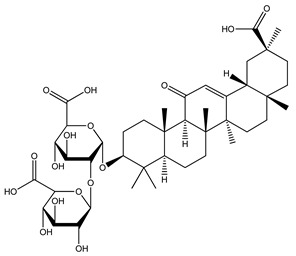
60. *Gymnema sylvestre*	Gymnemic acid	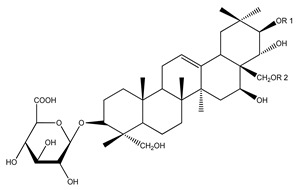
61. *Harungana madagascariensis*	Harunganin	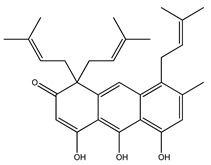
62. *Helicteres isora*	*p*-coumaric acid	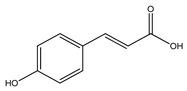
63. *Heritiera fomes*	Stigmasterol	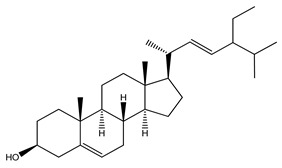
64. *Hibiscus esculentus*	Quercetin-3-*O*-gentiobioside	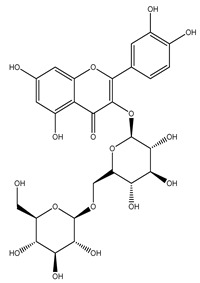
65. *Hibiscus rosa-sinensis*	Ascorbic acid	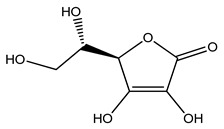
66. *Jatropha curcas*	Isoorientin	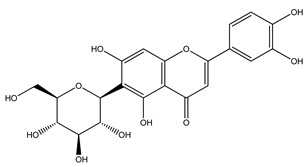
67. *Lantana camara*	Caffeic acid	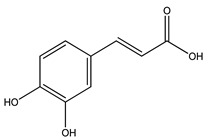
68. *Linum usitatissimum*	Ferulic acid	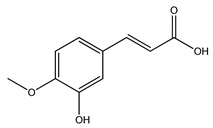
69. *Mangifera indica*	Mangiferin	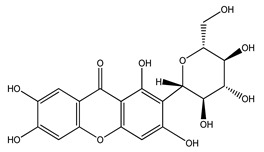
70. *Momordica charantia*	Vicine	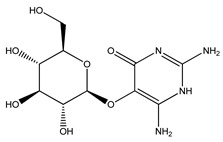
71. *Moringa oleifera*	Kaempferol	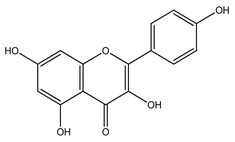
72. *Murraya koenigii*	Mahanimbine	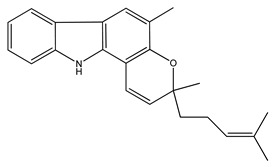
73. *Musa sapientum*	Delphinidin	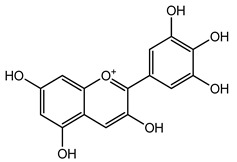
74. *Nigella sativa*	Thymoquinone	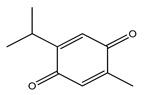
75. *Ocimum basicllicum*	Linalool	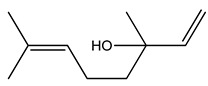
76. *Ocimum sanctum*	Eugenol	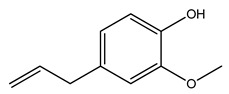
77. *Olea europaea*	Oleanolic acid	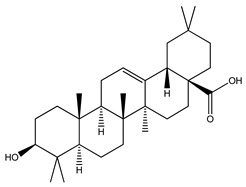
78. *Panax ginseng*	Ginsenoside Rg2	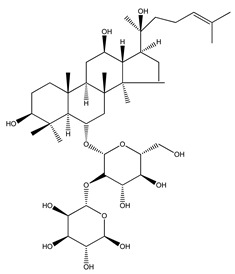
79. *Panda oleosa*	Ginsenoside Rb2	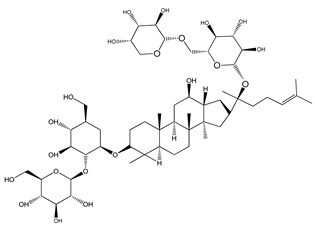
80. *Phaseolus vulgaris*	Orientin	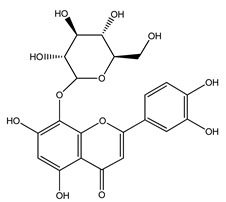
81. *Phyllanthus amarus*	Ursolic acid	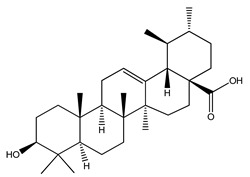
82. *Plantago ovata*	Myricetin	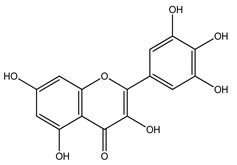
83. *Pterocarpus marsupium*	Marsupin	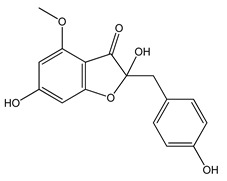
84. *Punica granatum*	Nictoflorin	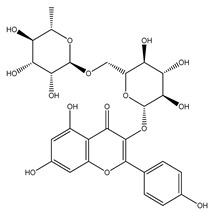
85. *Rehmannia glutinosa*	Catalpol	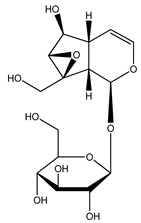
86. *Santalum album*	β-santalol	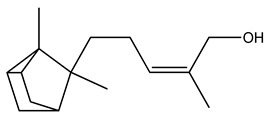
87. *Selaginella bryopteris*	Gallic acid	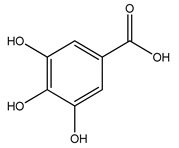
88. *Sesamum indicum*	Pinoresinol	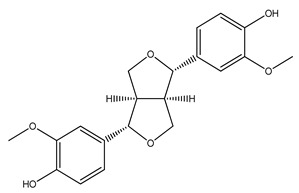
89. *Solanum nigrum*	Naringenin	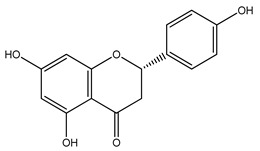
90. *Spirulina platensis*	β-carotene	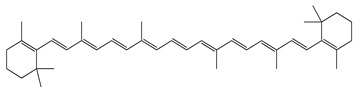
91. *Swertia chirayita*	Swerchirin	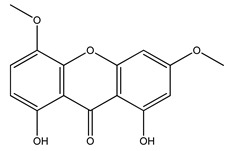
92. *Tamarindus indica*	Taxifolin	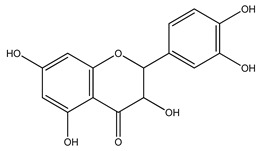
93. *Terminalia arjuna*	Arjungenin	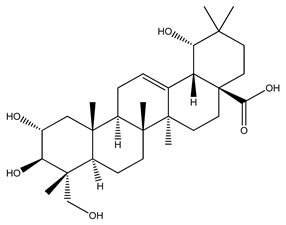
94. *Terminalia chebula*	Tannic acid	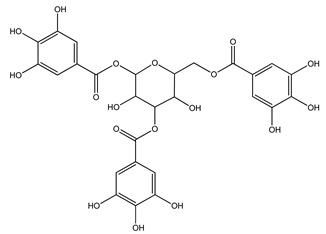
95. *Tinospora cordifolia*	Syringin	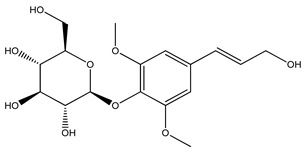
96. *Trigonella foenum-graecum*	Galactomannan	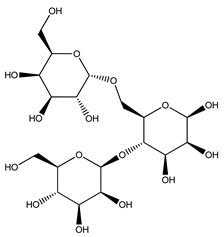
97. *Urtica dioica*	Quercitrin	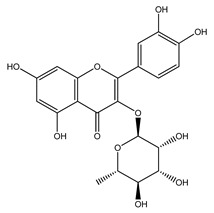
98. *Vernonia amygdalina*	Sobrerol	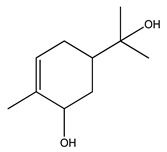
99. *Withania coagulans*	Withaferin A	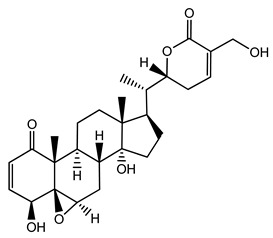
100. *Zingiber officinale*	Gingerol	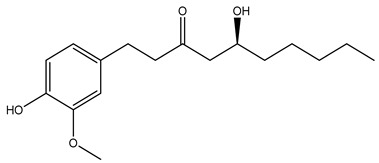

## Data Availability

Not applicable.

## Abbreviations

AMPK	5′ adenosine monophosphate-activated protein kinase
cAMP	cyclic Adenosine monophosphate
DPP-IV	Dipeptidyl peptidase-4
G6Pase	Glucose-6-phosphatase
GLP-1	Glucagon-like peptide-1
GLUT-2	Glucose transporter-2
GLUT-4	Glucose transporter-4
HbA1c	Hemoglobin A1c
IDF	International Diabetes Federation
K_ATP_	Adenosine triphosphate-sensitive potassium channel
PEPCK	Phosphoenolpyruvate carboxykinase
PI3K/AKT	Phosphoinositide 3-kinase/protein kinase B
PKA	Protein kinase A
PPAR-γ	Peroxisome proliferator-activated receptor-γ
SGLT	Sodium–glucose linked transporter
